# Higher Serum Uric Acid Is Associated with Higher Bone Mineral Density in Chinese Men with Type 2 Diabetes Mellitus

**DOI:** 10.1155/2016/2528956

**Published:** 2016-02-28

**Authors:** Dian-dian Zhao, Pei-lin Jiao, Jing-jia Yu, Xiao-jing Wang, Lin Zhao, Yan Xuan, Li-hao Sun, Bei Tao, Wei-qing Wang, Guang Ning, Jian-min Liu, Hong-yan Zhao

**Affiliations:** Department of Endocrinology and Metabolic Diseases, Ruijin Hospital, Shanghai Jiao-tong University School of Medicine, Shanghai Clinical Center for Endocrine and Metabolic Disease, Shanghai 310000, China

## Abstract

Accumulating evidence suggests that oxidative stress is associated with osteoporosis. Serum uric acid (UA) is a strong endogenous antioxidant. Therefore, we investigated the relationship between the serum UA and BMD in Chinese men with T2DM. In this cross-sectional study of 621 men with T2DM, BMDs at lumbar spine (L2–4), femoral neck (FN), and total hip (TH) were measured by dual-energy X-ray absorptiometry (DXA). Serum levels of UA, calcium (Ca), 25-OH vitamin D3 (vitD3), parathyroid hormone (PTH), and creatinine (Cr) were also tested. Data analyses revealed that serum UA levels were positively associated with BMD at all sites (*p* < 0.05) in men with T2DM after adjusting for multiple confounders. The serum UA levels were positively correlated with body weight (*r* = 0.322), body mass index (BMI) (*r* = 0.331), Ca (*r* = 0.179), and Cr (*r* = 0.239) (*p* < 0.001) and were also positively associated with the concentrations of PTH (*r* = 0.10, *p* < 0.05). When compared with those in the lowest tertile of UA levels, men with T2DM in the highest tertile had a lower prevalence of osteoporosis or osteopenia (adjusted odds ratio 0.54, 95% confidence interval [CI] 0.31–0.95). These data suggest that higher serum levels of UA are associated with higher BMDs and lower risks of osteoporosis in Chinese men with T2DM.

## 1. Introduction

Serum uric acid (UA) is produced from purines by xanthine oxidase and is considered a waste byproduct of human metabolism. In excess, UA may cause gouty arthritis, renal stones, or other illnesses [1–5]. In its crystalline state, UA can cause inflammatory reactions [6]. However, the role of dissolved UA is controversial. Increasing evidence suggests that higher serum UA levels within normal physiologic levels may have an advantageous effect due to its antioxidant properties [6–8]. The antioxidant effect of serum UA may potentially protect against metabolic bone diseases, such as osteoporosis.

Osteoporosis is a disease characterized by bone fragility and increased risk of fracture. Male osteoporosis is becoming recognized as an important public health concern, especially in male patients with type 2 diabetes mellitus (T2DM). Experimental, clinical, and epidemiologic evidence has shown that oxidative stress or low circulating levels of antioxidants have had adverse effects on bone metabolism; diabetes-related oxidative stress is one of the major predictors of bone loss [9, 10]. Recently, several clinical studies have shown that serum UA levels are correlated with bone mineral density (BMD) in men and postmenopausal women [11–14] and that higher serum UA levels act as protective factors against incident osteoporotic fractures in men [15]. However, whether this relationship is also present in men with T2DM has not been examined. In the present study, we examined associations between serum UA levels and BMD in Chinese men with T2DM.

## 2. Materials and Methods

### 2.1. Study Participants

This was a cross-sectional study. In this study, a total of 621 men with T2DM were enrolled from Ruijin Hospital, Shanghai Jiao-tong University School of Medicine. The T2DM diagnoses were consistent with the 1999 WHO diagnosis and classification criteria of diabetes mellitus. We excluded patients with type 1 diabetes mellitus (T1DM) and other special types of diabetes. Patients with diseases that could significantly affect bone metabolism, such as hypogonadism, Cushing syndrome, hyperthyroidism, hyperparathyroidism, and rheumatoid arthritis, were also excluded. Subjects with gout or urinary stones were also excluded. Patients with liver diseases or kidney diseases were likewise excluded. Patients who were taking drugs that could significantly affect bone metabolism, for example, Avandia, Alfacalcidol, Minodronic acid, Miacalcic, and Elcatonin were excluded. Finally, a total of 621 T2DM patients were included in the data analyses. This study was approved by the Institutional Review Board of Ruijin Hospital affiliated with Shanghai Jiao-tong University School of Medicine. Oral informed consent was obtained from each participant.

### 2.2. Clinical and Anthropometric Information

Interviews pertaining to sociodemographic characteristics, medication records, and any previous medical or surgical diseases were conducted by trained staff. Height was measured to the nearest 0.01 cm, and weight was recorded to the nearest 0.01 kg while participants were wearing lightweight clothing and no shoes. Body mass index (BMI) was calculated as body weight divided by height squared (kg/m^2^).

### 2.3. Biochemical Measurements

Patients were instructed to fast for at least 10 hours before morning blood collection. Fasting serum UA, calcium (Ca), and creatinine (Cr) levels were measured using an automatic biochemical analyzer (Modular E170, Roche, Basel, Switzerland). Serum levels of intact parathyroid hormone (PTH) were measured by intact immunoradiometric assay (reference range, 15–65 pg/mL; Abbott Diagnostics Division, Lake Forest, Illinois, USA). Serum levels of 25-OH vitamin D3 (vitD3) were measured by ECLIA, an electrochemiluminescence immunoassay (Cobas E601, Roche, Switzerland).

### 2.4. BMD Measurements

The BMDs at the lumbar spine (L2–4), femoral neck (FN), and total hip (TH) were all measured by dual energy X-ray absorptiometry (DXA, Lunar Expert-1313). According to the 1994 WHO recommended criteria, osteopenia is diagnosed by a −2.5 < *T*-score < −1.0 standard deviation (SD), and osteoporosis is diagnosed by a *T*-score < −2.5SD at any site on the lumbar spine, femoral neck, or total hip. In the present study, we classified subjects with osteopenia or osteoporosis as having “at least osteopenia (*T*-score < −1.0)”.

### 2.5. Statistics

All statistical analyses were carried out with SPSS 17.0. All normally distributed continuous variables were reported as the mean ± SD. The participants were divided into three groups according to the tertiles of UA: tertile 1: 0.065–0.305 mmol/L, tertile 2: 0.306–0.367 mmol/L, and tertile 3: 0.368–0.599 mmol/L. For comparison between groups of UA tertiles, ANOVAs for continuous variables were performed. A multiple linear regression analysis was performed to investigate the relationship between serum UA levels and BMD. A logistic regression was performed with the presence of at least osteopenia as a dependent variable. The accepted level of statistical significance was *p* < 0.05.

## 3. Results

The general characteristics of the study population are shown in Table 1. The average age was 54.14 ± 11.49 years (ranging from 19–84 years) and the mean serum UA level was 0.34 ± 0.08 mmol/L (ranging from 0.065–0.599 mmol/L). To better understand the clinical implications of the study, we categorized the subjects into three groups according to the serum UA concentrations (Table 1). Patients with higher serum UA levels were heavier and correspondingly had higher BMIs than those with lower UA concentrations, although the height in tertile 3 was slightly lower than that in tertile 2. The total serum Ca, PTH, and Cr levels were all significantly higher in the highest UA tertiles. However, there were no significant differences in age, VitD3, or diabetes duration among the groups. The BMD values at the L2–4, FN, and TH dose-dependently increased with the serum UA tertile level.

Multiple regression analyses were used to examine the associations between the serum UA levels and the BMD measurements at different skeletal sites. Estimates of fully adjusted regression models are presented in Table 2. Confounding factors, such as weight (*r* = 0.322, *p* < 0.001), height (*r* = 0.104, *p* = 0.01), BMI (*r* = 0.331, *p* < 0.001), and concentrations of serum Ca (*r* = 0.179, *p* < 0.001), PTH (*r* = 0.10, *p* = 0.021), and Cr (*r* = 0.239, *p* < 0.001) were all positively correlated with the serum UA levels. The serum UA levels were positively associated with the BMD values at the L2–4 (*r* = 0.216), FN (*r* = 0.176), and TH (*r* = 0.19) according to Pearson's correlation analyses (all *p* < 0.001). After adjusting for potential confounders in model 1 and model 2, the serum UA concentrations still demonstrated positive correlations with the BMDs at all sites (*R* ^2^ = 0.072 to 0.111; Table 2).

The overall proportion of patients who met the criteria for at least osteopenia was 33.5% (208/621). The proportions of at least osteopenia from the lowest UA tertile (T1) to the highest UA tertile (T3) were 42.0% (87/207), 34.8% (72/207) and 23.7% (49/207), respectively. After adjusting for potential confounders, multiple logistic regression analyses revealed that the odd ratios (ORs) for at least osteopenia dose-dependently increased from T3 to T1 (*p* for trend < 0.05; Table 3). In the fully adjusted model, after being adjusted for age, weight, height, diabetic duration, and serum concentrations of Cr, Ca, vitD3, and PTH, when compared with the patients in T1, the odds for at least osteopenia were 17% lower in T2 and 46% lower in T3.

## 4. Discussion

In the present study, we found that higher serum UA levels were associated with higher BMD in Chinese men with T2DM. The risk of osteoporosis or osteopenia significantly decreased by approximately 46% in patients in the highest tertile of UA. As far as we know, this is the first study to explore the association between serum UA levels and BMD in men with T2DM. Since Nabipour et al. [16] first reported a relationship between UA levels and bone health in older men, several other epidemiological studies have been performed. Whether in young and middle-aged males or in peri- and postmenopausal women, higher serum UA levels were all positively correlated with BMD, lower bone turnover, and lower prevalence of vertebra fracture. Thus, these studies and the present study consistently show that UA may have beneficial clinical effects on bone metabolism.

Oxidative stress has been identified as a potential mechanism that attenuates osteoblastogenesis and bone formation [17]. Reactive oxygen species (ROS) have been shown to inhibit the differentiation and proliferation of osteoblasts in vitro by regulating redox-sensitive signaling pathways [18]. ROS can improve bone resorption by directly stimulating the differentiation of osteoclasts or indirectly increasing the expression of ligands for NF-*κ*B, an osteoblast activator [19, 20]. UA may be involved in the pathogenesis of osteoporosis because of its antioxidant properties. Furthermore, serum UA competes with more than half of free radical scavenging activities in humans by quenching peroxides and singlet oxygen species [21]. The antioxidant properties of UA may help the human body resist aging, oxidative stress, and cell damage caused by oxidative stress, especially in myocardial cells, vascular smooth muscle cells, and nerve cells. Our study shows that higher UA levels result in higher bone mineral density and lower risks of osteoporosis or osteopenia in Chinese men with T2DM. This may be attributed to the antioxidant properties of UA. Ahn et al. [22] provided epidemiological evidence that serum UA may have beneficial effects on bone metabolism by acting as antioxidants in postmenopausal women. Moreover, the study showed that an in vitro UA treatment in mice significantly suppressed osteoclastogenesis in a dose-dependent manner and that UA can significantly decrease ROS contents in osteoclast precursors. However, further research is required to determine whether serum UA protects against oxidative stress-induced bone loss and the specific mechanism of this action.

Bone mineral density may be affected by a number of factors. In addition to the oxidative stress, BMD may be associated with renal function [23]. A recent study by Fujita indicated a significant inverse association between eGFR and BMD even after adjusting for age and BMI. As serum UA was associated with a more rapid decline of eGFR and incident renal insufficiency [24–26], serum UA may affect bone metabolism through renal function. The other possible mechanism that may explain the association between serum UA level and bone health is that the metabolic changes associated with osteoporosis influence the clearance of serum UA. Metabolic factors, such as serum calcium and PTH levels, may also influence the clearance of UA. In our study, serum UA levels were positively correlated with serum PTH and calcium concentrations. PTH is a hormone secreted from parathyroid glands that raises serum calcium levels by increasing absorption, reducing excretion, and promoting the release of calcium from bones. In turn, PTH is regulated by the serum calcium concentration. The renal handling of UA is partly dependent on PTH. A potential candidate site for this effect may be urate transporter 1, which reabsorbs the bulk of excreted UA from the glomerular ultrafiltrate [27]. In this study, serum Cr, PTH, and Ca levels were positively correlated with serum UA levels. However, even after adjusting for the serum Cr, PTH, and Ca levels, the relationship between serum UA levels and BMD remained, suggesting that the effect of UA on bone metabolism was not influenced by the serum Cr, PTH, Ca, and vitD3 levels.

There are a few limitations in this study. First, the study design was cross-sectional; that is, we could not determine whether a causal relationship existed between serum UA levels and osteoporosis-related phenotypes. Second, we controlled for a wide range of factors associated with serum UA levels. The results of this study may be subject to residual confounding. For example, we did not evaluate bone turnover markers (BTMs), which may affect BMD. Several studies reported a negative correlation between serum UA levels and BTMs [22, 27]. However, because we did not measure BTMs, we could not confirm these relationships. Third, our study population consisted of male patients selected only from Ruijin Hospital. Our study pool may not be representative of the general T2DM population and may also have a certain degree of bias. In addition, patients included in this study were divided into UA tertiles within the physiological range; whether this relation still exists remains to be revealed when the serum UA levels were above the upper limit of normal.

In conclusion, our study demonstrates that higher serum levels of UA were associated with higher BMDs and lower risks of osteoporosis or osteopenia in Chinese men with T2DM. Further experimental and longitudinal studies are necessary to clarify the association of serum UA levels and the development of osteoporosis in T2DM patients.

## Figures and Tables

**Table 1:** General characteristics of the study participants and comparison of characteristics according to serum UA tertiles.

	Total (*n* = 621)	Tertiles of serum uric acid level			*p*
		Tertile 1 (*n* = 207)	Tertile 2 (*n* = 207)	Tertile 3 (*n* = 207)	
Age (years)	54.14 ± 11.49	55.35 ± 11.60	53.22 ± 10.86	53.86 ± 11.93	0.155
Diabetes duration (years)	8.60 ± 6.45	8.86 ± 6.39	8.28 ± 6.46	8.67 ± 6.52	0.643
Height (cm)	171.94 ± 5.65	171.03 ± 5.83	172.43 ± 5.52	172.35 ± 5.50	0.018
Weight (kg)	74.35 ± 12.95	70.09 ± 11.38	74.78 ± 12.86	78.20 ± 13.28	<0.001
BMI (kg/m^2^)	25.08 ± 3.73	23.91 ± 3.34	25.09 ± 3.71	26.25 ± 3.77	<0.001
UA (mmol/L)	0.34 ± 0.08	0.26 ± 0.03	0.33 ± 0.02	0.42 ± 0.05	—
Ca (mmol/L)	2.21 ± 0.10	2.19 ± 0.12	2.22 ± 0.09	2.23 ± 0.10	0.002
PTH (pg/mL)	48.16 ± 19.75	45.21 ± 17.07	48.51 ± 19.71	50.75 ± 21.89	0.029
Cr (*μ*mol/L)	75.57 ± 20.31	70.25 ± 18.74	74.93 ± 15.33	81.55 ± 24.31	<0.001
vitD3 (nmol/L)	38.21 ± 15.33	38.57 ± 14.34	36.59 ± 15.63	39.42 ± 15.98	0.223
BMD					
L2–4 (g/cm^2^)	1.20 ± 0.19	1.17 ± 0.18	1.21 ± 0.19	1.24 ± 0.18	0.001
FN (g/cm^2^)	0.95 ± 0.14	0.92 ± 0.12	0.95 ± 0.14	0.97 ± 0.14	<0.001
TH (g/cm^2^)	1.01 ± 0.14	0.98 ± 0.13	1.02 ± 0.14	1.04 ± 0.15	<0.001

Serum uric acid tertiles were as follows: tertile 1, 0.065–0.305 mmol/L; tertile 2, 0.306–0.367 mmol/L; tertile 3, 0.368–0.599 mmol/L. BMI: body mass index; UA: uric acid; Ca: serum calcium; PTH: parathyroid hormone; Cr: serum creatinine; vitD3: 25-OH vitamin D3; BMD: bone mineral density; L2–4: lumbar spine 2–4; FN: femoral neck; TH: total hip.

**Table 2:** Multiple linear regression analysis of the relationship between the serum UA levels and BMD.

BMD (g/cm^2^)	Model 1				Model 2			
	*B*	SE	*p*	Adjusted *R* ^2^	*B*	SE	*p*	Adjusted *R* ^2^
Lumbar spine 2–4	0.410	0.102	<0.001	0.086	0.415	0.126	0.001	0.084
Femoral neck	0.196	0.074	0.008	0.111	0.190	0.092	0.040	0.098
Total hip	0.201	0.076	0.009	0.093	0.192	0.095	0.044	0.072

Model 1: adjusted for age, weight, height, and diabetic duration; Model 2: adjusted for factors listed in Model 1 in addition to serum concentrations of creatinine, calcium, vitD3, and parathyroid hormone.

**Table 3:** The risk of osteoporosis in relation to tertiles of serum UA.

	Tertile 1 (*n* = 207)	Tertile 2 (*n* = 207)	Tertile 3 (*n* = 207)	*p* for trend
Osteoporosis/osteopenia				
Model 1	1	0.74 (0.49–1.09)	0.43 (0.28–0.65)	<0.0001
Model 2	1	0.88 (0.58–1.33)	0.56 (0.36–0.87)	0.011
Model 3	1	0.83 (0.50–1.36)	0.54 (0.31–0.95)	0.033

Odds ratios (ORs) and 95% confidence intervals (CI) for osteoporosis and osteopenia according to the serum uric acid (UA) tertiles after adjusting for confounders.

Model 1: unadjusted.

Model 2 is adjusted for age, weight, height, and diabetic duration.

Model 3 is further adjusted for factors listed in Model 1 in addition to serum concentrations of creatinine, calcium, vitD3, and parathyroid hormone.
